# Quasi‐Saturated Layer: Implications for Estimating Recharge and Groundwater Modeling

**DOI:** 10.1111/gwat.12916

**Published:** 2019-07-05

**Authors:** Roger D. Gonçalves, Elias H. Teramoto, Bruno Z. Engelbrecht, Miguel A. Alfaro Soto, Hung K. Chang, Martinus Th. van Genuchten

**Affiliations:** ^1^ Center for Environmental Studies and Basin Studies Laboratory São Paulo State University, UNESP Rio Claro SP 13506‐900 Brazil.; ^2^ Department of Applied Geology São Paulo State University, UNESP Rio Claro SP 13506‐900 Brazil.; ^3^ Department of Nuclear Engineering Federal University of Rio de Janeiro, COPPE Rio de Janeiro RJ 21941‐972 Brazil.

## Abstract

This study presents an extension of the concept of “quasi‐saturation” to a quasi‐saturated layer, defined as the uppermost dynamic portion of the saturated zone subject to water table fluctuations. Entrapped air here may cause substantial reductions in the hydraulic conductivity (*K*) and fillable pore water. Air entrapment is caused by a rising water table, usually as a result of groundwater recharge. The most significant effects of entrapped air are recharge overestimation based on methods that use specific yield (*S*_y_), such as the water table fluctuation method (WTF), and reductions in *K* values. These effects impact estimation of fluid flow velocities and contaminant migration rates in groundwater. In order to quantify actual groundwater recharge rates and the effects of entrapped air, numerical simulations with the FEFLOW (Version 7.0) groundwater flow model were carried out using a quasi‐saturated layer for a pilot area in Rio Claro, Brazil. The calculated recharge rate represented 16% of the average precipitation over an 8‐year period, approximately half of estimates using the WTF method. Air entrapment amounted to a fillable porosity of 0.07, significant lower that the value of 0.17 obtained experimentally for *S*_y_. Numerical results showed that the entrapped air volume in the quasi‐saturated layer can be very significant (0.58 of the air fraction) and hence can significantly affect estimates of groundwater recharge and groundwater flow rates near the water table.

## Abstract 2

*Article impact statement*: Quasi‐saturation due to air entrapment near the water table has major effects on estimated recharge rates and modeling groundwater.

## Introduction

The upward movement of a water table (WT) in response to groundwater recharge causes a displacement of air (the nonwetting phase) by water (the wetting‐phase) in partially saturated vadose zone pores. Many studies have shown that a significant amount of air can become entrapped in the largest pores below the WT during this ascension (e.g., Smith and Browning 1943; Christiansen 1944; Debacker 1967; Tokunaga and Narasimhan 1987; Faybishenko 1995; Fry et al. 1997; Williams and Oostrom 2000; Sakaguchi et al. 2005; Marinas et al. 2013). The presence of entrapped air promotes considerable but reversible changes in the aquifer properties. The term “quasi‐saturated zone” was introduced by Faybishenko (1995) to describe the uppermost portion of the saturated zone partially filled with entrapped air. Although containing water and air in varying proportions, the quasi‐saturated layer is distinct from the capillary fringe in that the pressure head is positive, similar to a fully saturated zone, and not negative like in the vadose zone.

Several studies have focused on reductions in the saturated hydraulic conductivity (*K*) by air entrapment (Smith and Browning 1943; Christiansen 1944; Orlob and Radhakrishna 1958; Debacker 1967; Faybishenko 1995; Fry et al. 1997; Sakaguchi et al. 2005; Zlotnik et al. 2007; Marinas et al. 2013). The range of *K* reductions due to pore clogging by entrapped air has been shown to be very broad. For instance, Zlotnik et al. (2007) verified a reduction of 50% due to entrapped air, whereas Faybishenko (1995) observed reductions of one to two orders of magnitude. They used the term quasi‐saturated hydraulic conductivity (hereafter denoted by *K*_quasi_) for the resulting conductivity.

Several authors, including Peck (1969), Constantz et al. (1988), and Fry et al. (1997), showed that the amount of entrapped air depends on soil type and the prevailing grain size distribution, as well as on the fluid flow rate (Constantz et al. 1988; Fry et al. 1997), moisture content (Fayer and Hillel 1986b), and pore geometry (Peck 1969; Fry et al. 1997). Furthermore, numerous experiments have shown a broad range of measured entrapped air saturation contents, between approximately 0.04 for clays and 0.55 for coarse sands (Christiansen 1944; Fayer and Hillel 1986a; Stonestrom and Rubin 1989; Faybishenko 1995; Wang et al. 1998; Sakaguchi et al. 2005; Marinas et al. 2013).

Due to WT fluctuations (WTFs), entrapped air is subject to changes in hydraulic pressures, which results in the compression or expansion of entrapped air bubbles (Christiansen 1944; Gupta and Swartzendruber 1964; Collis‐George and Yates 1990; Bicalho et al. 2005; Marinas et al. 2013). Marinas et al. (2013) observed reductions between 18% and 26% in the amount of entrapped air at a water pressure of 2.5 m, compared to zero pressure, with the amount of entrapped air decreasing approximately linearly with the increase in water pressure.

Entrapped air will obstruct parts of otherwise saturated pores below the WT and as such can significantly reduce local fluid flow velocities and estimated groundwater recharge rates (Christiansen 1944; Constantz et al. 1988; Faybishenko 1995; Heilweil et al. 2004). Regarding shallow unconfined aquifers, the actual porosity filled by water during a rising WT (the fillable porosity, *θ*_f_) is often smaller than the specific yield (*S*_y_) due to entrapped air below the WT (Kayane 1983; Maréchal et al. 2006). As a consequence of this discrepancy, recharge rates calculated using the WTF method (Healy and Cook 2002) will likely be overestimated since calculated values are very sensitive to the assumed value of *S*_y_.

Considering the potential impact of entrapped air on calculated WTFs, especially in shallow unconfined aquifers, this work aims to introduce the concept of a “quasi‐saturated layer” as based on several earlier studies, as well as on field evidence. The applicability of the quasi‐saturated concept to practical problems is illustrated by computing more realistic groundwater recharge fluxes using the FEFLOW groundwater flow simulator of Diersch (2014) as applied to an unconfined aquifer in Rio Claro, Brazil.

## Conceptual Model of the Quasi‐Saturated Layer

**Figure 1:**
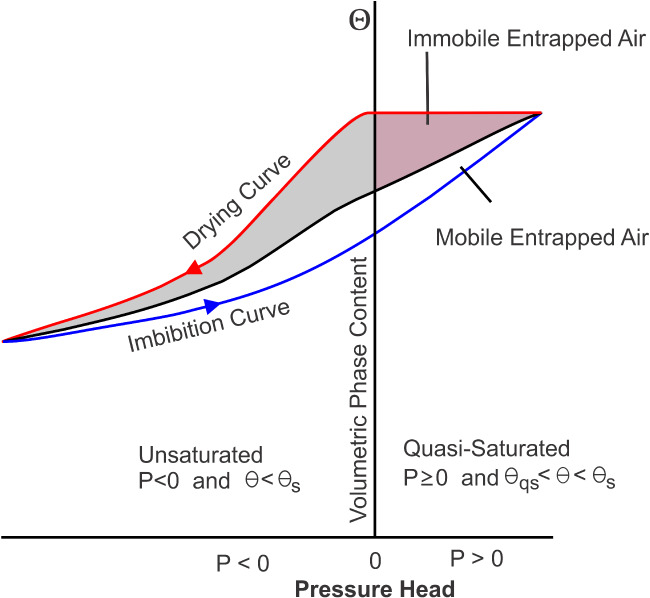
Conceptual model illustrating the mechanism of air entrapment due to hysteresis involving primary drainage and imbibition WRCs (after Faybishenko 1995).

Figure 1 provides a schematic of the effects of air entrapment during an imbibition event on the water retention curve (WRC), when water invades the fillable pore space and air bubbles become entrapped by snap‐off and bypassing mechanisms. Faybishenko (1995) suggested that entrapped air at and near the WT is distributed into mobile and immobile parts. While the mobile part can be displaced, entrapped air remains as an immobile and entrapped phase within the pores, and is responsible for a hysteresis effect during alternating cycles of fluid drainage and imbibition.

**Figure 2:**
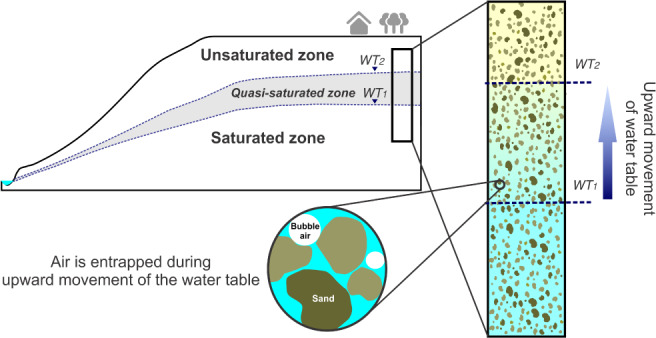
Schematic showing how during recharge the water table rises from WT1 to WT2. The presence of significant proportions of entrapped air reduces the hydraulic conductivity and fillable porosity.

In our study we view the upper part of the aquifer subject to air entrapment as a separate layer between the vadose zone and the fully saturated zone, all in the vicinity of a fluctuating WT. Although the quasi‐saturated zone is ephemeral and dissipates when the WT moves down, its recurrent formation when the WT moves up (Figure 2) allows one to consider this zone as a separate (seasonal) layer of the aquifer. Moreover, entrapped gases within this zone may persist for a decade or longer, even without WTFs at this depth (Ryan et al. 2000; McLeod et al. 2015). Although the pore system is filled with different proportions of air and water, the main feature distinguishing the quasi‐saturated zone from the capillary fringe is that the quasi‐saturated zone has a positive pressure head, similar to the fully saturated zone. Moreover, the hydraulic properties (*K*_quasi_ and *θ*_f_) of the quasi‐saturated zone are controlled by the volume of air entrapped in the aquifer pores.

**Figure 3:**
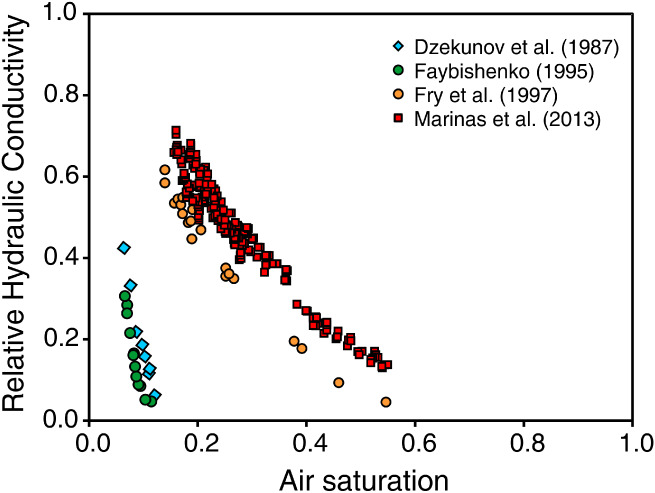
Relative hydraulic conductivity (*K*_rel_ = *K*_quasi_/*K*_*s*_) of quasi‐saturated soils as a function of entrapped air saturation.

Table 1 presents results from laboratory tests related to entrapped air saturations in various porous media. Air saturation as used here is the volumetric entrapped air content divided by the porosity. The estimated values exhibit a wide range that may reach relative air saturation values above 0.50. Several studies (e.g., Constantz et al. 1988; Marinas et al. 2013) have noted that materials with large pores, such as sands and well‐aggregated soils, tend to have higher entrapped air proportions than media with finer pore structures. Laboratory tests by Dzekunov et al. (1987), Faybishenko (1995), and Fry et al. (1997), on the other hand, showed that fine‐textured media produced more pronounced reductions in *K* than coarse‐grained soils with increasing entrapped air saturation (Figure 3).

**Table 1:** Entrapped Air Fractions Obtained Experimentally

References	Medium	Proportion of Pore Space Filled with Entrapped Air
Poulovassilis (1970)	Glass beads	0.19
Stonestrom and Rubin (1989)	Coarse sand	0.126
Christiansen (1944)	Coarse sand	0.15 to 0.40
Wang et al. (1998)	Coarse sand	0.154 to 0.305
Marinas et al. (2013)	Fine to coarse sands	0.130 to 0.545
Williams and Oostrom (2000)	Fine sand	0.15
Sakaguchi et al. (2005)	Sandy loam	0.23
Sakaguchi et al. (2005)	Aggregated clay	0.13
Fayer and Hillel (1986a)	Loamy sand	0.043 to 0.126
Stonestrom and Rubin (1989)	Loam	0.069
Faybishenko (1995)	Loams	0.10 to 0.25

Some of the experimental data further revealed an approximately exponential decrease in the hydraulic conductivity with increasing entrapped air saturation (Figure 3). This relationship can be described also by a power law as proposed previously by Faybishenko (1995) for loam soils:

(1) $$K_{\text{quasi}}=K_{0}+\left(K_{s}-K_{0}\right){\left(1-\frac{\omega }{\omega_{\max}}\right)}^{n}$$

where *K*_quasi_ is the quasi‐saturated hydraulic conductivity; *K*_0_ is the minimum quasi‐saturated hydraulic conductivity; *K*_S_ is the saturated hydraulic conductivity without air entrapment; *ω* is the volumetric fraction of entrapped air; *ω*_max_ is the maximum entrapped air content; and *n* an exponent. The fillable porosity hence is related to specific yield (*S*_y_) through

(2) $$\theta_{f}=S_{y}\left(1-\omega \right)$$

These findings are consistent with the general shape of the unsaturated hydraulic conductivity as a function of water content, with or without the presence of entrapped air (Luckner et al. 1989), as shown by Carsel and Parrish (1988) among others for the van Genuchten (1980) unsaturated soil hydraulic conductivity function. Although Fry et al. (1997) reported the applicability of the van Genuchten model for quasi‐saturated systems, Marinas et al. (2013) noted that the Faybishenko equation equally or better captured the hydraulic conductivity changes for their data.

The vertical zone where the WT fluctuates periodically can be identified using monitoring wells or other means. That zone is considered here to be a distinct and seasonal layer with reductions in the fillable porosity (*θ*_f_ < *S*_y_) and hydraulic conductivity (*K*_quasi_ < *K*_s_) due to entrapped air. This separate layer is referred to here as the quasi‐saturated layer. Instead of assuming variably saturated conditions typical for the vadose (unsaturated) zone with its negative pressure heads, this layer can be simulated using aquifer conditions with positive hydraulic pressures.

## Study Site

**Figure 4:**
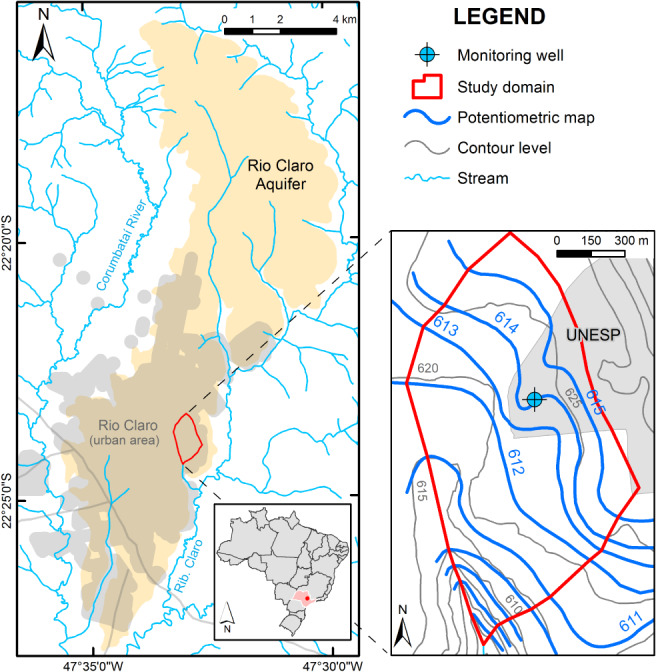
Location of the study area and monitoring well (left) and a potentiometric map (Gonçalves 2016) showing also the topography with elevation in meters above mean sea level.

The study site is located at São Paulo State University in the city of Rio Claro, Brazil (Figure 4). The site is part of the Rio Claro Aquifer, which has been studied and monitored extensively since 2000 (e.g., Ferreira and Caetano‐Chang 2008; Neto et al. 2016; Gonçalves and Chang 2018). The Rio Claro Aquifer is a shallow unconfined aquifer composed of Cenozoic sedimentary rocks of the Rio Claro Formation overlaying the Permian aquitard of the Corumbataí Formation. The aquifer covers approximately 85 km^2^ on top of the Paraná Basin, with its thickness varying from a few meters to up to 50 m (about 15 m at the study location). Sediments have fluvial origin and are composed mostly of fine‐ to medium‐grained sands with a variable clay matrix (Gonçalves and Chang 2018).

**Figure 5:**
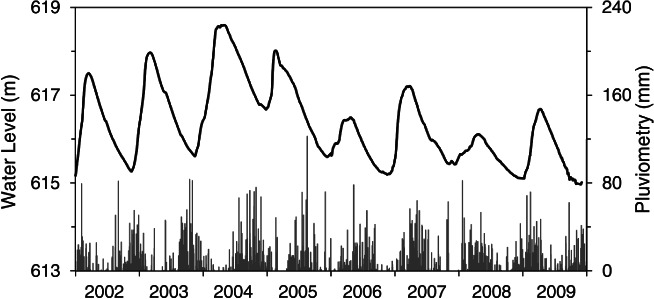
Monitored groundwater levels (black solid line) showing the seasonal cycles of recharge and drawdown as well as daily rainfall rates (gray bars).

The Rio Claro Aquifer presents a plateau morphology with recharge occurring in most of the aquifer at relatively high topographic locations, and discharge along streams that cross the aquifer with their bottom near the lower contact. Lateral hydraulic gradients at the site are very low, less than 0.007, because of its location near a groundwater divide and functioning as an important recharge zone. The WT generally varies between depths of about 6 to 10 m, but with a large seasonal variation due the occurrence of alternating seasons of rainy summers and dry winters (Gonçalves 2016). Hydraulic conductivities determined by slug tests ranged from 2.0 × 10^−6^ m/s to 2.0 × 10^−4^ m/s (Gonçalves and Chang 2018), while a value of 0.17 for *S*_y_ of the saturated soils was obtained by analyzing the WRCs of undisturbed samples (Alfaro Soto and Chang 2008). The precipitation data (Figure 5) were obtained from daily total rainfall records provided by the meteorological station of the Center for Environmental Studies and Planning at UNESP, located in the study area.

## Numerical Model

A three‐dimensional saturated flow model was constructed to simulate transient WTFs in an unconfined aquifer. We used for our simulations the FEFLOW (version 7.0) finite‐element code of Diersch (2014), but modified to include the effects of air entrapment during groundwater recharge periods. The model domain of 1.1 km^2^ was divided into 60,944 finite elements (30,807 nodes) with irregular spacing both in the horizontal and vertical directions. The domain was divided into two layers with different hydraulic properties to reflect the existence of entrapped air. The upper layer was a quasi‐saturated layer, defined by the vertical range where the WT was found to fluctuate periodically, while the lower layer represented the fully saturated zone.

**Figure 6:**
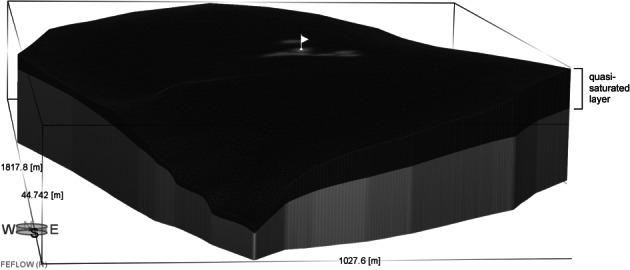
Three‐dimensional (3D) view of the finite‐element model setup.

The adopted configuration can be seen in Figure 6. The domain bottom is the contact with the aquitard, and hence was considered to be no‐flow boundary, similarly as the northern and eastern limits which represented groundwater divides. In addition, seepage face boundary conditions were introduced along the southeastern border to reproduce discharge areas, thus allowing free outflow from the model. Recharge rates were represented using a transient fluid‐flux boundary condition along the top elements. Values of the saturated hydraulic conductivity (*K*_s_, as estimated from slug tests), and of *S*_y_ (derived from water retention data) were applied to the lower layer, whereas *K*_quasi_ and *θ*_f_ values were calculated for the quasi‐saturated layer considering the entrapped air effects.

Monitored groundwater levels from 2002 to 2005 (1460 d) were used on a daily basis in order to calibrate the transient model. The initial condition of the hydraulic head for the transient simulations was obtained using a single calibrated steady‐state run, while the bottom surface of the top layer was approximated by means of a steady‐state run for the lowest WT period. Once the calculations satisfactorily represented the transient hydraulic head data for the first 4 years, an additional 4‐year simulation was carried out and compared with the monitored hydraulic heads from 2006 to 2009.

## Numerical Validation of the Quasi‐Saturated Layer

In order to estimate the entrapped air content, a set of transient‐state simulations was performed representing the two main layers of the conceptual model: a quasi‐saturated layer on top of a fully saturated zone. For *S*_y_ we used a value of 0.17 as estimated from measured water retention data, and for *K*_s_ of the laterally homogeneous saturated lower layer a value of 2 × 10^−5^ m/s, being the mean value as derived from slug tests Due to air entrapment, the hydraulic parameters (*K*_*quasi*_ and *θ*_*f*_) of the quasi‐saturated layer were variable since they depend on air saturation.

The grain size distributions of the sands tested by Marinas et al. (2013) were very similar to those of the Rio Claro Formation sediments (Gonçalves and Chang 2018). Because of this similarity, we employed an exponential function (rather than the van Genuchten hydraulic function) fitted to the experimental data, with *K*_quasi_ being a function of the entrapped air content. The following exponential function was fitted to the Marinas et al. (2013) data shown in Figure 3:

(3) $$K_{\text{quasi}}=1.314\;K_{s}\;e^{-3.969\omega }$$

**Figure 7:**
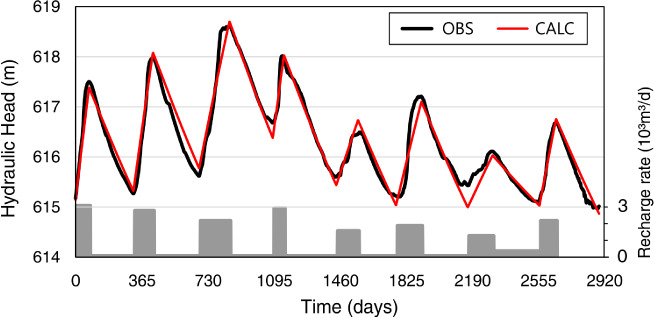
Comparison between observed (black solid line) and simulated (red solid line) water levels for an 8‐year‐long simulation using the quasi‐saturated layer. Also represented as gray bars are the estimated recharge rates during each recharge period.

We calibrated the numerical model using observed transient WT data over a 4‐year period as shown in Figure 7. The model could be run using a daily time step. Adjustable parameters were the volumetric fraction of entrapped air, *ω*, in Equations 2 and 3, and the recharge rate. The recharge rate was assumed to be uniform laterally, and constant in time during each ascending and descending WTF stage (Figure 7), without any constraints on its values. A relatively high entrapped air fraction (0.58) was obtained, leading to values of 2.5 × 10^−6^ m/s for *K*_quasi_ (Equation 3) and 0.07 for *θ*_f_ (Equation 2) of the quasi‐saturated layer. Calculated hydraulic heads showed very good correlation with the observed data as reflected by a root mean square error (RMSE) of 0.0545 m and a coefficient of determination (*R* ^2^) of 0.96. Table 2 lists the calculated recharge rates.

**Table 2:** Calculated Recharge Rates for the Time Period 2002 through 2009 Assuming a Quasi‐Saturated Layer, and Observed Annual Rainfall Rates (with Their Ratio of Precipitation)

Year	Accumulated Precipitation (mm)	Calculated Recharge (mm/year; % of Precipitation)
2002	1026	203 (19.8%)
2003	1463	265 (18.1%)
2004	1820	321 (17.6%)
2005	1248	161 (12.9%)
2006	1514	164 (10.9%)
2007	1365	229 (16.8%)
2008	1318	174 (13.2%)
2009	1317	222 (16.9%)

Figure 7 shows considerable variability in time of the observed and calculated heads, which we initially thought would have affected the value of θ_*f*_ during the calculations. We found, however, that it was not necessary to vary θ_*f*_ in time to properly calibrate the heads. Calibrated recharge rates were found to be significantly lower than those previously estimated using the WTF method (Neto and Chang 2008). The average calculated recharge for the 2002 to 2005 period was 249 mm/year, in contrast to 491 mm/year as obtained using the WTF method. The recharge rates were also estimated by Neto et al. (2016) by simulating infiltration and variably saturated flow in the soil profile, including root water uptake, using the Richards equation. They found an average value of 304 mm/year, which is much closer to the value of 249 mm/year we obtained.

In order to confirm the reliability of the quasi‐saturated model, an additional 4 years were simulated using the calibrated model without further changes in the hydraulic parameters, including of θ_*f*_, except for setting the recharge rates as listed in Table 2. The results in Figure 7 show excellent agreement of the predicted values with the observed WTFs, having a RMSE of 0.0497 m, and a *R*^2^ of 0.96.

## Discussion

Effective porosities derived from laboratory experiments often lead to inaccurate predictions of the upward and downward movement of a shallow WT in transient groundwater models. Unrealistically large groundwater recharge rates may then be needed to simulate observed WT peaks correctly. Laboratory estimates of the porosity may similarly lead to poor predictions when the WT moves downward. These difficulties may be resolved by using lower hydraulic conductivities and effective porosities resulting from air entrapment at and below the WT.

Our study shows the importance of focusing on the prevailing mechanisms causing lower hydraulic conductivities and effective porosities. Several recent studies (e.g., Rama et al. 2018; Teramoto and Chang 2018) found that specific yield values calibrated using transient models were significantly lower than expected for the lithologies involved, or as estimated from the soil water retention properties. Teramoto and Chang (2018) considered this deviation as a hysteresis effect, while Rama et al. (2018) assumed the upper portion of the aquifer to remain unsaturated, thus using the Richards equation in conjunction with van Genuchten (1980) type soil hydraulic functions to calculate the WTFs. Those two studies, as well as numerical simulations carried out by Nogueira and Chang (2015) and those presented in this paper, all required reductions in the fillable porosity to properly reproduce observed WTFs.

The decrease in the hydraulic conductivity and the effective porosity of the quasi‐saturated domain could be described also using van Genuchten expressions for the unsaturated hydraulic conductivity as a function of air or fluid saturation (van Genuchten 1980; Luckner et al. 1989; Fry et al. 1997). As demonstrated by Marinas et al. (2013), and further used in our study, the original equation by Faybishenko (1995) performed equally well. We also note here a separate study by Neto et al. (2016) who used the Richards equation for flow in variably saturated vadose systems to simulate the WTFs and to estimate recharge rates at the same site of the Rio Claro Aquifer for the period from 2002 to 2005. Groundwater recharge was well estimated on average (304 mm/year). However, their model was not capable to properly predict the transient WTFs. Moreover, their approach needed far more data (including specific soil hydraulic and root water uptake parameters), and is numerically far more complex than a fully saturated model. For these reasons we believe that the use of a quasi‐saturated layer in groundwater flow simulations as used in our study can be extremely beneficial and opportune.

Several previous studies have shown that air entrapment can be significant and durable in the uppermost portion of the saturated zone. Calculations using the WTF method for this reason tend to overestimate recharge rates, especially for shallow unconfined aquifers. This finding has a major impact on water resources management since water resource estimates are commonly sensitive to the assumed effective porosity, as well as for evaluating alternative groundwater exploitation scenarios, estimating fluid flow velocities and/or contaminant migration rates, and predicting the long‐term effects of climate change.

In order to evaluate the effects of air entrapment effects, and to moderate numerical simulation errors, experimental tests should be conducted more systematically to estimate the air entrapment fraction during rising WTs under different geological settings. Based on the obtained entrapped air estimates, we strongly suggest a review and correction of recharge rates calculated using the WTF method. Hence, for correct recharge calculations that also consider entrapped air, the fillable porosity term (*θ*_f_) instead of *S*_y_ may need to be used (i.e., *R* = *θ*_f_ Δ*h*/Δ*t*, where *R* is the recharge rate, *h* is the WT height, and *t* is time).

Despite the robustness of our numerical model calibration, certain aspects remain unanswered. Nonuniqueness continues to be a prominent nature of groundwater models, and a major source of error in recharge predictions by model calibration. Calibration may assure the potential feasibility of a conceptual model, but does not necessarily account for model singularity since calibration may be obtained with different model parameterizations. For example, Knowling and Werner (2017) demonstrated that distinct combinations of *S*_y_, the recharge rate and *K*_s_ can produce diverse goodness of fits of long‐term time series of water level data. This reflects the difficulty of quantifying time‐varying groundwater recharge rates through groundwater model calibration. Indeed, the favorable calibration of our model using WT data is not sufficient to prove the key role of entrapped air in estimating groundwater recharge. Still, our model suggests the critical role of air entrapment controlling anomalous WTFs in field‐scale problems, and may explain the inability of calibrations employing laboratory derived *S*_y_ values. Since entrapped air and pore clogging leads to noticeable reductions in *S*_y_ and *K*_S_, it represents another source of uncertainty to reliable quantification of groundwater recharge. In summary, the quasi‐saturated layer encompasses a phenomenological approach that can improve the performance of groundwater flow models, particularly for unconfined aquifers.

Finally, we note that our study focused on the physical effects of air entrapment in terms of groundwater modeling, WT dynamics, and estimating recharge. The effects of air entrapment, however, are not limited to the physical effects of fluid flow at and below the WT. Air entrapment in the uppermost part of the saturated zone, as well as in the capillary fringe, is conducive to several physical, biological, and geochemical processes. To exemplify, the concentration of noble gases below the WT at levels above equilibrium with atmospheric air (also known as “excess air”) have been linked experimentally and numerically to entrapped air, in which the gases are transferred from entrapped air to groundwater by diffusional movement (Heaton and Vogel 1981; Aeschbach‐Hertig et al. 2000, 2008; Holocher et al. 2003; Mächler et al. 2013). Likewise, Williams and Oostrom (2000), Mächler et al. (2013), Mcleod et al. (2015), and Teramoto and Chang (2019) demonstrated that the presence of entrapped air delivers oxygen to groundwater. Aquifer oxygenation by entrapped air dissolution can have important implications to biogeochemical process in the saturated zone. For example, Teramoto and Chang (2019) showed that entrapped air may be the most important cause of aquifer oxygenation by driving the oxidation of byproducts (such as CH_4_ and Fe^2+^) and affecting the redox state of hydrocarbon‐contaminated aquifers. For these reasons the possible physical, microbial, and geochemical effects of air entrapment should not be neglected when studying the seasonal movement of groundwater in the near surface.

## Conclusions

The exact effects of air entrapment on recharge calculations are uncertain and still being investigated since it is very difficult, if not impossible, to assess these effects directly in field conditions, despite the numerous laboratory and field experiments that have been carried out over the years (Faybishenko 1997). The proposed groundwater model using a quasi‐saturated layer was capable to satisfactorily predict WTFs observed under field conditions considering air entrapment effects on the hydraulic parameters in the upper portion of the aquifer. Recharge rates calculated using the WTF method potentially may be overestimated significantly, which is especially critical when the fillable porosity is much lower than the specific yield due to high entrapped air fractions. Our study shows that transient groundwater flow modeling using a quasi‐saturated layer is a very effective approach for predicting WTFs and estimating transient recharge distributions in shallow unconfined aquifers.
